# Numerical Analysis of Physical Characteristics and Heat Transfer Decoupling Behavior in Bypass Coupling Variable Polarity Plasma Arc

**DOI:** 10.3390/ma15093174

**Published:** 2022-04-27

**Authors:** Fan Jiang, Qi Miao, Bin Xu, Shinichi Tashiro, Manabu Tanaka, Sanbao Lin, Chenglei Fan, Shujun Chen

**Affiliations:** 1 Engineering Research Center of Advanced Manufacturing Technology for Automotive Components, Beijing University of Technology, Ministry of Education, Beijing 100124, China; jiangfan@bjut.edu.cn (F.J.); 18852894619@139.com (Q.M.); sjchen@bjut.edu.cn (S.C.); 2 State Key Laboratory of Advanced Welding and Joining, Harbin Institute of Technology, Harbin 150001, China; sblin@hit.edu.cn (S.L.); fclwh@hit.edu.cn (C.F.); 3 Joining and Welding Research Institute, Osaka University, Osaka 5670047, Japan; tashiro@jwri.osaka-u.ac.jp (S.T.); tanaka@jwri.osaka-u.ac.jp (M.T.)

**Keywords:** bypass coupling VPPA, numerical analysis, physical characteristics, decoupled heat transfer

## Abstract

A novel bypass coupling variable polarity plasma arc was proposed to achieve the accurate adjusting of heat and mass transfer in the welding and additive manufacturing of aluminum alloy. However, the physical characteristics and decoupled transfer behavior remain unclear, restricting its application and development. A three-dimensional model of the bypass coupling variable polarity plasma arc was built based on Kirchhoff’s law, the main arc and the bypass arc are coupled by an electromagnetic field. The model of current attachment on the tungsten electrode surface is included for simulating different heating processes of the EP and EN phases in the coupling arc. The distribution of temperature field, flow field, and current density of the bypass coupling variable polarity plasma arc was studied by the three-dimensional numerical model. The heat input on the base metal under different current conditions is quantified. To verify the model, the arc voltages are compared and the results in simulation and experiment agree with each other well. The results show that the radius of the bypass coupling arc with or without bypass current action on the base metal is different, and the flow vector of the bypass coupling arc plasma with bypass current is larger than the arc without bypass current. By comparing the heat transfer on the electrodes’ boundary under different current conditions, it is found that increasing the bypass current results in the rise in heat input on the base metal. Therefore, it is concluded that using bypass current is unable to completely decouple the wire melting and the heat input of the base metal. The decoupled degree of heat transfer is one of the important factors for accurate control in the manufacturing process with this coupling arc.

## 1. Introduction

Wire arc additive manufacturing has a great prospect in the field of additive manufacturing due to its advantages, including low cost and high efficiency [1]. However, there are also some problems, such as low forming accuracy and a coarse microstructure [2,3], which can be attributed to improper control of the heat input of the base metal. It is found that a faster cooling rate leads to smaller grain size, and more controllable heat input leads to better performance of formed parts and higher forming accuracy [4,5]. Normally, the melting speed of the wire and the deposition efficiency are reduced synchronously to reduce the heat input of the base metal, which means that there is a strong coupling relationship between the cladding rate and the heat input [6,7,8]. This is a big challenge for the accurate control when using arc heat source in additive manufacturing. At present, heat sources of arc additive manufacturing mainly include the gas tungsten arc [9] (GTA), gas mater arc [10] (GMA), plasma arc [11] (PA), cold metal transfer [12] (CMT), and some hybrid arcs. To separately adjust the energy distribution on the wire and the base metal, Zhang et.al. [13,14,15,16] proposed the bypass coupling arc to decouple mass and heat transfer. The bypass GTA was established between the wire and the tungsten electrode, which make it possible to keep the base metal heat input while increasing the deposition rate.

According to this principle, Huang et.al. [17,18] proposed bypass microbeam plasma arc additive manufacturing and studied the dynamic behavior of the droplet on the molten pool during the stationary pileup process. It was found that, with the increase in the number of droplets entering the molten pool, both the bath height and the melting width increased; however, the penetration depth was almost constant. Shi Yu et.al. [19,20,21] proposed the efficient welding process of the double-wire bypass coupled arc GMAW and studied the droplet transition behavior of the welding process. The results showed that the different welding current waveform and the matching form would lead to the change of the forces exerted on the welding droplet. Yang et.al. [22,23] proposed a GMA additive manufacturing process with the tungsten inert gas arc as the bypass and analyzed the forming characteristics of thin-wall steel parts. It was found that the width of the deposited multi-layer parts decreased with the increasing of the bypass current, and the deposited height increased in proportion to the bypass current under the same deposition rate.

Even though the bypass coupling arc form is designed by its theoretical decoupling characteristic of heat and mass transfer, the arc physics and decoupling ability of this novel method is still not clear. The current of the main arc and bypass arc both flow through the same tungsten electrode whose state is one of the dominant factors for the arc properties. Whichever current changes, the tungsten electrode states such as temperature and electron emission change, thus, the arc output changes. It shows that the two arcs have combined but still have a certain degree of mutual influence while decoupling the heat and mass transfer, which makes the accurate control of manufacturing very complex. For applicating this arc form in an aluminum alloy and making the additive manufacturing more stable and accurate [24,25,26], the variable polarity plasma arc (VPPA) has been used as the main arc, and the bypass coupling VPPA is proposed in this study. The main arc and bypass arc both worked in variable polarity current waveform, which make the heat and mass transfer decoupling more flexible, but the physical process is more complicated. Therefore, to clarify the physics and decoupling ability of heat transfer in the bypass coupling VPPA is necessary and helpful for the process parameters to be accurately designed. It is difficult to study the physical characteristics of the bypass coupling arc by experimental means, and the heat transfer between the arc and the electrode cannot be accurately quantified, so this paper uses the experimental principles to establish a numerical simulation model to study it, so as to study its decoupling behavior more in-depth.

In this work, a three-dimensional model of the bypass coupling variable polarity plasma arc was built based on Kirchhoff’s law, the main arc and the bypass arc are coupled by electromagnetic field. The temperature distribution of different cross sections in VPPA and the coupling arc were compared to study the interaction between the bypass arc and the main arc. To understand the decoupling ability of heat transfer in bypass coupling VPPA, the heat transfer between the arc and the electrodes under different current conditions was compared. By combining the distribution of physical field and the energy transfer between the arc and the electrodes, the heat transfer mechanism of the novel process was discussed. Finally, the accuracy of the simulation model was verified by experiments. This paper was divided into five sections. Section 1 introduces issues about the bypass coupling arc. The numerical simulation model and the experimental verification are presented in Section 2. The simulation results and discussion are described in Section 3. Section 4 concludes the findings of this study.

## 2. Principle and Model of Heat Source

### 2.1. Principle of Heat Source

**Figure 1:**
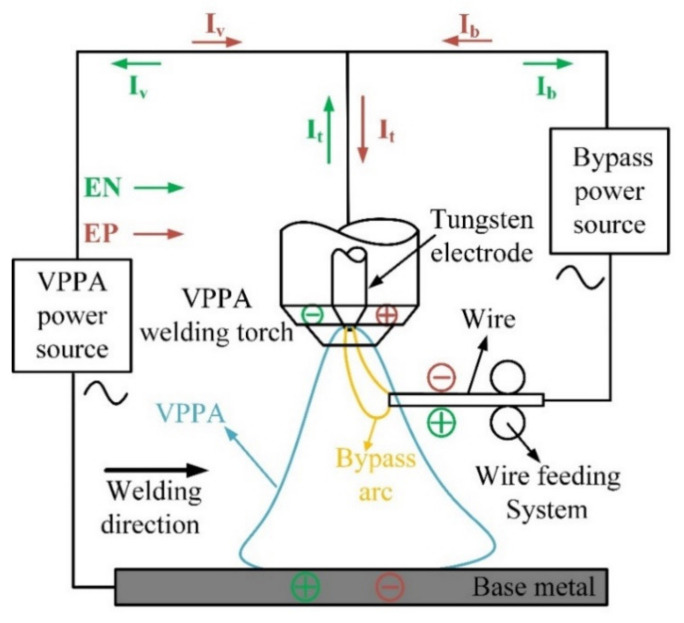
Schematic diagram of bypass coupled variable polarity arc process.

The principle of the bypass coupling variable polarity plasma arc is shown in Figure 1, which is mainly composed of two variable polarity welding power sources, the VPPA welding torch and the bypass wire feeding mechanism. The VPPA, as the main arc, is established between the tungsten electrode and the base metal. The bypass arc is established between the tungsten electrode and the wire. The two arcs are controlled by two welding power sources separately. The sum of welding current is I_t_ (the current on the tungsten electrode). After I_t_ flows out of the tungsten electrode, it is divided into I_b_ and I_v_. I_b_ (the current on the wire) flows to the bypass arc to produce heat and melt the welding wire. I_v_ (the current on the base metal) flows through the base metal to heat and melt the base metal.

**Figure 2:**
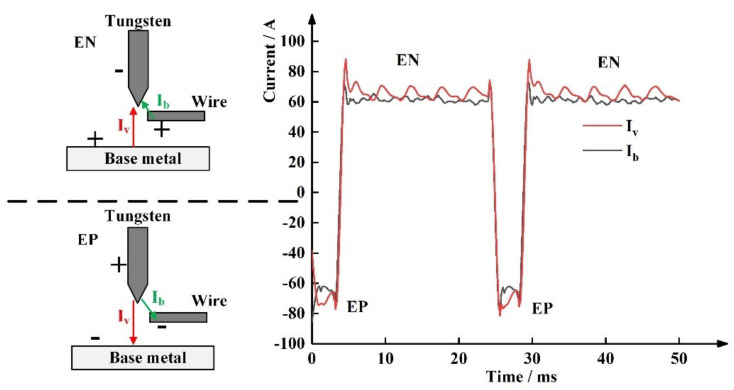
Current waveform diagram.

Figure 2 shows the schematic diagram of arc morphology with different polarity and the current waveform of the coupling arc obtained in the experiment. In the electrode negative (EN) phase, the polarity of the tungsten electrode is negative, and the polarity of the base metal and the wire is positive. The values of I_v_ and I_b_ are positive. In the electrode positive (EP) phase, the polarity of the tungsten electrode is positive, and the polarity of the base metal and the wire is negative. The values of I_v_ and I_b_ are negative.

### 2.2. Calculated Domain

**Figure 3:**
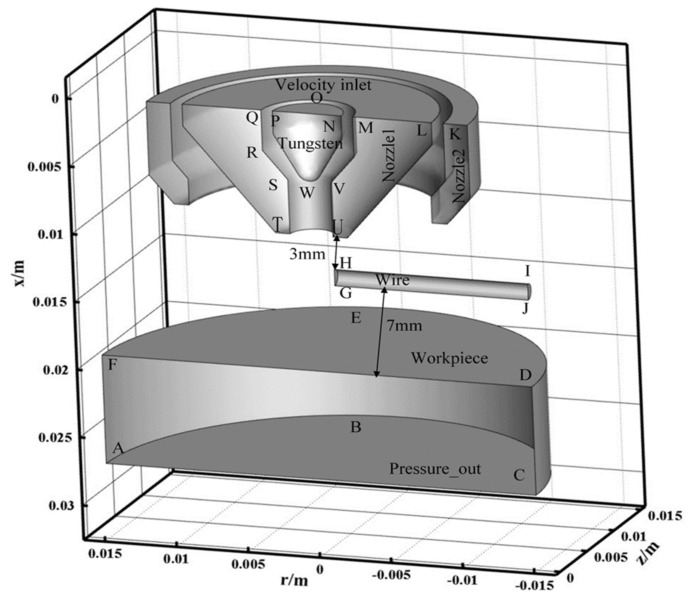
Computational domain diagram.

Figure 3 is the calculation domain. The tungsten, the nozzle, the base metal, and the wire are the solid region, and the rest is the fluid region. The base metal and the wire are set as solid to study the physical mechanism of energy transfer without melting. The diameter of the tungsten electrode is 4.8 mm, the setback of the tungsten electrode is 4 mm, and the diameter of the wire is 4.8 mm. The length of TU is 3.2 mm. The flow rate of the plasma gas is 2.0 L/min, and the flow rate of the shielding gas is 15 L/min. Both the plasma gas and shielding gas are argon. The material of the wire is set as 5A06 aluminum. The material of the base metal is set as 5A06 aluminum. As shown in Table 1, 5A06 aluminum properties and parameters are given. The potential on both the base metal and the wire is 0 V. The potential on the tungsten electrode is set by the user-defined function. As shown in Table 2, the boundary conditions are given according to the actual manufacturing process.

### 2.3. Simulation Conditions

The simulation conditions are the same as that used in the experiments, as shown in Table 3. The distance from TU to FD is 10 mm. The energy transfer between the arc and the electrodes under different current conditions is obtained by setting six groups of different current values. The six groups of different current values can be realized by setting the current density of the tungsten electrode and the wire. By the comparison of the results, the decoupling degree of heat and mass transfer in this process is analyzed.

### 2.4. Magnetic Fluid Dynamics Model

The following assumptions are proposed: (1) the plasma arc is in a state of local thermodynamic equilibrium; (2) the viscous dissipation is ignored; (3) the plasma arc is axially distributed in the computational domain; (4) the plasma arc is a continuous medium. Based on these assumptions, the governing equations are proposed.

Mass conservation equation:

(1) $$\frac{\partial \rho }{\partial t}+\rho \mathrm{div}v=0$$

Momentum conservation equation:

(2) $$\begin{array}{c} \frac{\partial (\rho u)}{\partial t}+\rho div\left(uv\right)\\ =J_{y}B_{z}-J_{z}B_{y}-\frac{\partial P}{\partial x}+\frac{\partial }{\partial x}\left\{\mu \left[2\frac{\partial u}{\partial x}-\frac{2}{3}\left(\frac{\partial u}{\partial x}+\frac{\partial v}{\partial y}+\frac{\partial w}{\partial z}\right)\right]\right\}\\ +\frac{\partial }{\partial y}\left[\mu \left(\frac{\partial u}{\partial y}+\frac{\partial v}{\partial x}\right)\right]+\frac{\partial }{\partial z}\left[\mu \left(\frac{\partial w}{\partial x}+\frac{\partial u}{\partial z}\right)\right] \end{array}$$

(3) $$\begin{array}{c} \frac{\partial \left(\rho v\right)}{\partial t}+\rho div\left(vv\right)\\ =J_{z}B_{x}-J_{x}B_{z}-\frac{\partial P}{\partial y}+\frac{\partial }{\partial y}\left\{\mu \left[2\frac{\partial v}{\partial y}-\frac{2}{3}\left(\frac{\partial u}{\partial x}+\frac{\partial v}{\partial y}+\frac{\partial w}{\partial z}\right)\right]\right\}\\ +\frac{\partial }{\partial z}\left[\mu \left(\frac{\partial v}{\partial z}+\frac{\partial w}{\partial y}\right)\right]+\frac{\partial }{\partial x}\left[\mu \left(\frac{\partial v}{\partial x}+\frac{\partial u}{\partial y}\right)\right] \end{array}$$

(4) $$\begin{array}{c} \frac{\partial \left(\rho w\right)}{\partial t}+\rho div\left(wv\right)\\ =J_{x}B_{y}-J_{y}B_{x}-\frac{\partial P}{\partial z}+\frac{\partial }{\partial z}\left\{\mu \left[2\frac{\partial w}{\partial z}-\frac{2}{3}\left(\frac{\partial u}{\partial x}+\frac{\partial v}{\partial y}+\frac{\partial w}{\partial z}\right)\right]\right\}\\ +\frac{\partial }{\partial x}\left[\mu \left(\frac{\partial w}{\partial x}+\frac{\partial u}{\partial z}\right)\right]+\frac{\partial }{\partial y}\left[\mu \left(\frac{\partial w}{\partial y}+\frac{\partial v}{\partial z}\right)\right] \end{array}$$

Energy conservation equation:

(5) $$\begin{array}{c} \frac{\partial \left(\rho c_{p}T\right)}{\partial t}+\rho c_{p}\mathrm{div}\left(Tv\right)\\ \;\;\;\;\;\;\;\;\;\;\;\;\;\;\;\;\;\;\;\;\;\;\;\;=\frac{\partial }{\partial x}\left(\frac{k}{c_{p}}\frac{\partial T}{\partial x}\right)+\frac{\partial }{\partial y}\left(\frac{k}{c_{p}}\frac{\partial T}{\partial y}\right)+\frac{\partial }{\partial z}\left(\frac{k}{c_{p}}\frac{\partial T}{\partial z}\right)+\frac{J_{x}^{2}+J_{y}^{2}+J_{z}^{2}}{\sigma }-S_{R} \end{array}$$

In type, ρ is density, *t* is time, *u*, *v*, *w* are velocity components in three directions, respectively, *J*_x_, *J_y_*, *J_z_* are current density of three directions, *B_x_*, *B_y_*, *B_z_* are magnetic induction intensity in three directions, respectively, P is pressure, *μ* is the viscosity coefficient, *c_p_* is specific heat, *k* is thermal conductivity, *k_B_* is the Boltzmann constant, the value of *k_B_* is 1.380649 × 10^−23^ J/K, *S_R_* is radiation loss.

The Lorenz force term in the momentum conservation equation and the Joule heating term in the energy conservation equation needs to solve the distribution of magnetic induction intensity B and current density J; hence, it is needed to solve Max’s equations.

Ohm’s law:

(6) $$J_{x}=-\sigma \frac{\partial \phi }{\partial x},J_{y}=-\sigma \frac{\partial \phi }{\partial y},J_{z}=-\sigma \frac{\partial \phi }{\partial z}$$

Current continuity equation:

(7) $$\frac{\partial }{\partial x}\left(\sigma \frac{\partial \phi }{\partial x}\right)+\frac{\partial }{\partial y}\left(\sigma \frac{\partial \phi }{\partial y}\right)+\frac{\partial }{\partial z}\left(\sigma \frac{\partial \phi }{\partial z}\right)=0$$

Poisson equation of magnetic vector potential:

(8) $$-\nabla^{2}A_{x}=\mu_{0}J_{x},-\nabla^{2}A_{y}=\mu_{0}J_{y},-\nabla^{2}A_{z}=\mu_{0}J_{z}$$

(9) $$B_{x}=\frac{\partial A_{z}}{\partial y}-\frac{\partial A_{y}}{\partial z},B_{y}=\frac{\partial A_{x}}{\partial z}-\frac{\partial A_{z}}{\partial x},B_{z}=\frac{\partial A_{y}}{\partial x}-\frac{\partial A_{x}}{\partial y}$$

In type, σ is conductivity, $A_{x}$, $A_{y}$, $A_{z}$ are the magnetic vectors of three directions, and $\mu_{0}$ is the vacuum permeability.

To simulate the VPPA welding process, it is necessary to simulate the heat at the interface between the plasma arc and the electrode. An additional energy source term is required for the tungsten electrode and the base metal. At the EN and EP phase, the tungsten electrode and the base metal are the electron emission electrodes, respectively. The tungsten is a thermal cathode and the base metal is a cold cathode, which means that the heat transfer process on the tungsten surface and the base metal surface is different.

At the interface between the plasma arc and the tungsten, the additional source term of the cathode at the EN phase includes thermionic electron emission cooling, ion recombination heating, and radiation cooling:

(10) $$H_{tk}=\left|j_{i}\right|V_{i}-\left|j_{e}\right|\varphi_{k}-\varepsilon \alpha T^{4}$$

At the EP phase, the additional source term includes electron heating and radiation cooling:

(11) $$H_{ta}=\left|j\right|\varphi_{a}-\varepsilon \alpha T^{4}$$

At the interface between the plasma arc and the base metal, in the EN phase, the additional source term is the same as that of tungsten surface in the EP phase:

(12) $$H_{ba}=\left|j\right|\varphi_{a}-\varepsilon \alpha T^{4}$$

In the EP phase, the additional source term is the same as that of the tungsten surface in the EN phase:

(13) $$H_{bk}=\left|j_{i}\right|V_{i}-\left|j_{e}\right|\varphi_{k}-\varepsilon \alpha T^{4}$$

In the above equations, ε is the surface emissivity, α is the Stefan–Boltzmann constant, the value of α is 5.67 × 10^−8^ W × (m^−2^·K^−4^), T is the temperature, $\varphi_{k}$ is the work function of the cathode, $\varphi_{a}$ is the work function of the anode, *V_i_* is the ionization potential of argon, $j_{e}$ is the electron current density, $j_{i}$ is the ion current density, and $\left|j\right|=\left|j_{e}\right|+\left|j_{i}\right|$ is the total current density at the cathode surface calculated from the current continuity equation.

In above equations, the key problem is to define current densities of the electron and ion. Generally, the Richardson current density theory is used to separate $j_{e}$ and $j_{i}$ if the tungsten electrode is the cathode. The value of $j_{e}$ cannot exceed the Richardson current density, which gives an upper limit of the electron current density by thermionic electron emission:

(14) $$\left|j_{R}\right|=A_{c}T^{2}\exp\left(-\frac{e\varphi_{e}}{K_{B}T}\right)$$

where $A_{c}$ is the thermionic emission constant for the cathode surface and $\varphi_{e}$ is the effective work function for thermionic emission of the electrode surface at the local surface temperature. For ThO_2_, the value of $A_{c}$ is 5. For La_2_O_3_, the value of $A_{c}$ is 96. For Ce_2_O_3_, the value of $A_{c}$ is 30. The value of $j_{i}$ is then assumed to be $\left|j\right|-$ $\left|j_{R}\right|\;$if $\left|j\right|$ is greater than $\left|j_{R}\right|$.

In the EP phase, the base metal and the wire emits electrons. The base metal and the wire are the cold cathode, which means that it is not suitable for the Richardson theory of current density. This also suggests that the electron current dominates the total current density of the arc, rather than the ionic current. Therefore, the values of $j_{e}$ and $j_{i}$ were set to be 50% and 50% of the total current density, respectively, when the base metal is the cathode. In addition, considering the temperature evolution on the tungsten electrode surface when the polarity changes, the simplified current attachment model for the tungsten electrode suggested by Tanaka et al. [27] is coupled with the MHD model.

### 2.5. Model of Current Attachment on the Tungsten Electrode Surface

In the EN phase, the tungsten electrode is heated to a high temperature as a hot cathode. The thermal electron emission capability of the tungsten electrode is high. In the EP phase, the base metal and the wire are not heated to a high temperature like the cold cathode. The thermal electron emission capability is very poor. The model of current attachment on the tungsten electrode surface is added to describe the thermal electron emission capability of the tungsten electrode at different temperatures. The simplified current attachment model of the tungsten electrode proposed by Tanaka et al. [27] is coupled with the MHD model described in Section 3.1. The model has been shown to express the current attachment on the tungsten electrode by adding small amounts of emitter material (such as ThO_2_, La_2_O_3_, and Ce_2_O_3_) in TIG welding. In this model, the emitter material is 2% ThO_2_, which is the same as that used in the experiment.

**Figure 4:**
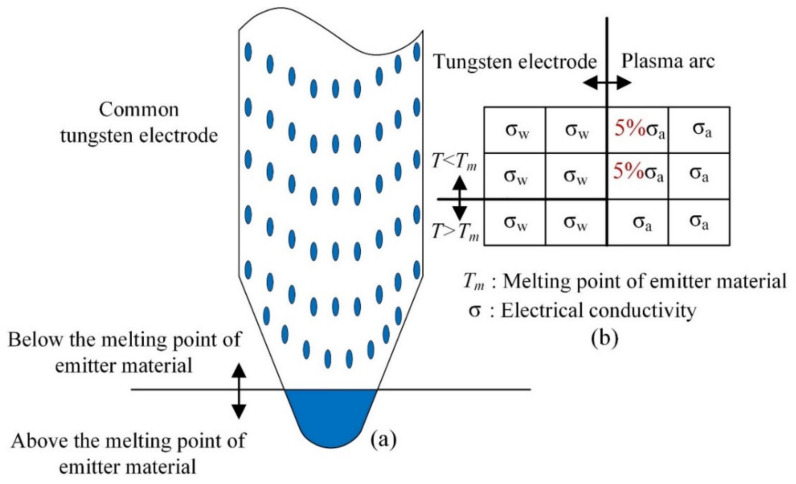
(**a**) Schematic diagram of the current attachment on the tungsten electrode, (**b**) treatment of current attachment.

The current attachment characteristics of the thermionic cathode are concluded by Tanaka et al. [28]. As shown in Figure 4a, emitter material below the melting point is dispersed over the surface of tungsten. For simplification, we assume that the thermionic electron emission of the emitter material is replaced with the electrical conductivity of the arc plasma on the cathode surface [29]. As shown in Figure 4b, by comparing the cathode surface temperature with the melting point of the emitter material, the electrical conductivity of the arc plasma on the cathode surface is assumed [30]. The flux of the arc current from one grid to another grid is represented by *I* = S(σE), where S is the area between two grids. We assume to replace 5% S by 5% σ.

**Figure 5:**
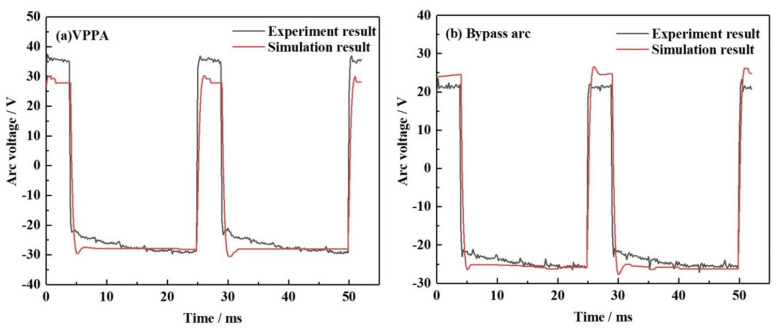
Voltage signals of numerical simulation and experiment: (**a**) VPPA, (**b**) bypass arc.

Figure 5 shows the measured voltage waveform of numerical simulation and experiment in two periods. The parameters used in the numerical simulation are the same as that used in the experiment. The value of the VPPA current and bypass current are both 60 A, and the value of the current at different polarities is equal. Figure 5a shows the VPPA voltage waveform in the numerical simulation and experiment, respectively. The experimental voltage waveform is roughly similar with the simulated data shown in the figures. Figure 5b shows the bypass arc voltage waveform in the numerical simulation and experiment, respectively. The experimental voltage waveform is roughly similar with the simulated data shown in the figures. The simulated voltage output waveforms are in good agreement with the experimental results, which verifies the correctness of the simulation model.

## 3. Results and Discussion

According to the established numerical calculation model of the bypass coupling variable polarity plasma arc, the user-defined equation (UDF) is written. The transient calculation method is used to calculate the bypass coupling variable polarity plasma arc in Fluent software, and the corresponding time of the following results in the EP and EN phases is t = 0.004 s and t = 0.02 s. The physics of the bypass coupling variable polarity plasma arc are calculated.

### 3.1. The Physics of Bypass Coupling Variable Polarity Plasma Arc

**Figure 6:**
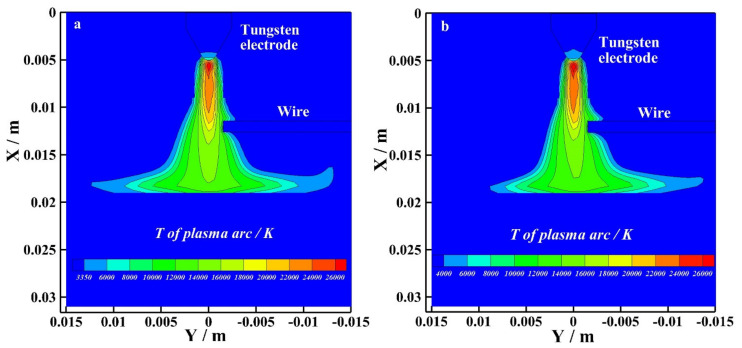
The temperature distribution at EP phase with and without bypass current, (**a**) 120 A and 0 A, (**b**) 60 A and 60 A.

Figure 6 shows the temperature distribution of the bypass coupling arc at the EP phase with and without bypass current. As shown in Figure 6a, I_v_ =120 A and I_b_ = 0 A. The wire exists, but the bypass arc does not exist. The maximum temperature of the bypass coupling arc reaches 26,000 K at the EP phase. As shown in Figure 6b, I_v_ = 60 A and I_b_ = 60 A. The bypass arc exists. The maximum temperature of the bypass coupling arc also reaches 26,000 K at the EP phase. By observing the temperature distribution below the tungsten electrode, the temperature distribution of the arc with and without bypass current are basically the same. By observing the temperature distribution below the wire and the radius of arc action on the base metal, the radius of arc action on the base metal with bypass current is smaller than that without bypass current.

**Figure 7:**
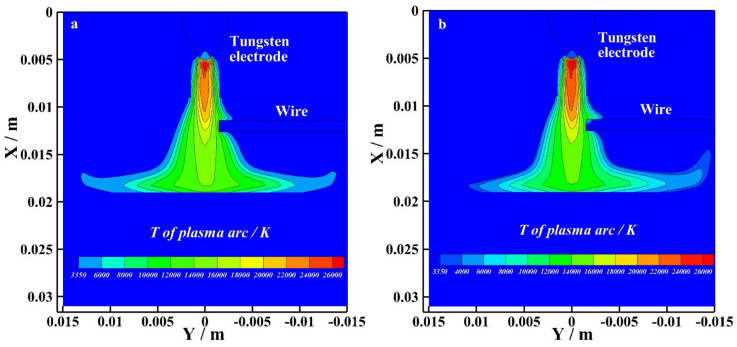
The temperature distribution at EN phase with and without bypass current, (**a**) 120 A and 0 A, (**b**) 60 A and 60 A.

Figure 7 shows the temperature distribution of the bypass coupling arc at the EN phase with and without bypass current. As shown in Figure 7a, I_v_ = 120 A and I_b_ = 0 A. The wire exists, but the bypass arc does not exist. The maximum temperature of the bypass coupling arc reaches 26,000 K at the EP phase. As shown in Figure 7b, I_v_ = 60 A and I_b_ = 60 A. The bypass arc exists. The maximum temperature of the bypass coupling arc also reaches 26,000 K at the EP phase. By observing the temperature distribution below the tungsten electrode, the temperature distribution of the arc with and without bypass current are basically the same. By observing the temperature distribution below the wire and the radius of arc action on the base metal, the radius of the bypass coupling arc with bypass current action on the base metal is smaller.

**Figure 8:**
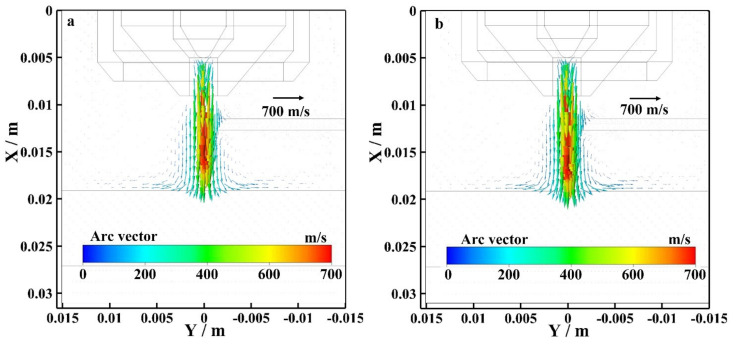
Flow field distribution of bypass coupling arc at EP phase with and without bypass current, (**a**) 120 A and 0 A, (**b**) 60 A and 60 A.

Figure 8 shows the flow vector of the bypass coupling arc plasma at the EP phase with and without bypass current. As shown in Figure 8a, I_v_ = 120 A and I_b_ = 0 A. The wire exists, but the bypass arc does not exist. The maximum flow rate is mainly concentrated on the plasma arc axis, and the maximum flow rate is 700 m/s. As shown in Figure 8b, I_v_ = 60 A and I_b_ = 60 A. The bypass arc exists. The maximum flow rate is mainly concentrated on the plasma arc axis, and the maximum flow rate is also 700 m/s. By observing the arc flow vector distribution near the tungsten electrode, the flow vector distribution of the arc with and without bypass current are basically the same. By observing the flow vector distribution of the arc plasma along the axis, the flow vector of the bypass coupling arc plasma with bypass current is larger than the arc without bypass current at EP.

**Figure 9:**
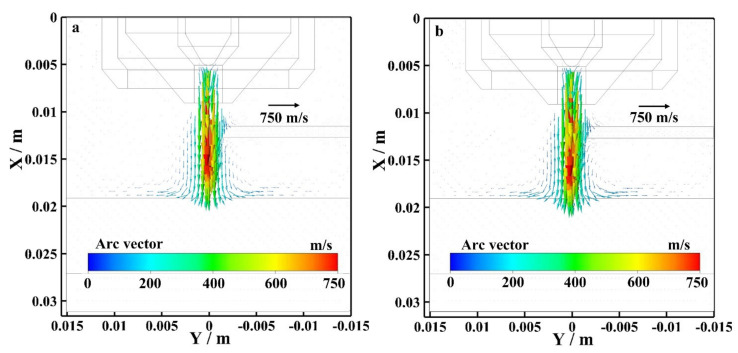
Flow field distribution of bypass coupling arc at EN phase with and without bypass current, (**a**) 120 A and 0 A, (**b**) 60 A and 60 A.

Figure 9 shows the flow vector of the bypass coupling arc plasma at the EN phase with and without bypass current. As shown in Figure 9a, I_v_ = 120 A and I_b_ = 0 A. The wire exists, but the bypass arc does not exist. The maximum flow rate is mainly concentrated on the plasma arc axis, and the maximum flow rate is 750 m/s. As shown in Figure 9b, I_v_ = 60 A and I_b_ = 60 A. The bypass arc exists. The maximum flow rate is mainly concentrated on the plasma arc axis, and the maximum flow rate is also 750 m/s. By observing the arc flow vector distribution near the tungsten electrode, the flow vector distribution of the arc with and without bypass current are basically the same. By observing the flow vector distribution of the arc plasma along the axis, the flow vector of the bypass coupling arc plasma with bypass current is larger than the arc without bypass current at EN.

**Figure 10:**
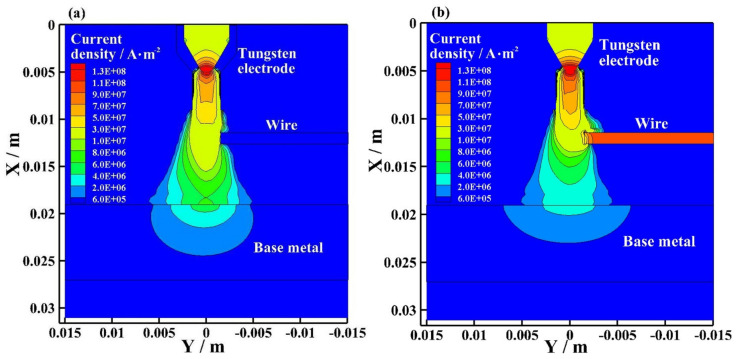
Current density distribution of bypass coupling arc at EP phase with and without bypass current, (**a**) 120 A and 0 A, (**b**) 60 A and 60 A.

Figure 10 shows the current density distribution of the bypass coupling arc at the EP phase with and without bypass current. As shown in Figure 10a, I_v_ = 120 A and I_b_ = 0 A. The wire exists, but the bypass arc does not exist. The maximum current density appears near the tip of the tungsten electrode. There is no current density distribution on the wire. The current density distribution is mainly concentrated on the plasma arc axis, and the maximum current density is 1.30 × 10^8^ A·m^−^^2^. As shown in Figure 10b, I_v_ = 60 A and I_b_ = 60 A. The bypass arc exists. The maximum current density appears near the tip of the tungsten electrode, and the maximum current density is 1.30 × 10^8^ A·m^−2^. The maximum current density on the wire is approximately 1.10 × 10^8^ A·m. In the bypass coupling arc with bypass curent, the current density distribution near the wire is more concentrated than the arc without bypass current at EP. The current density distribution on the base metal is smaller than the arc without bypass current at EP.

**Figure 11:**
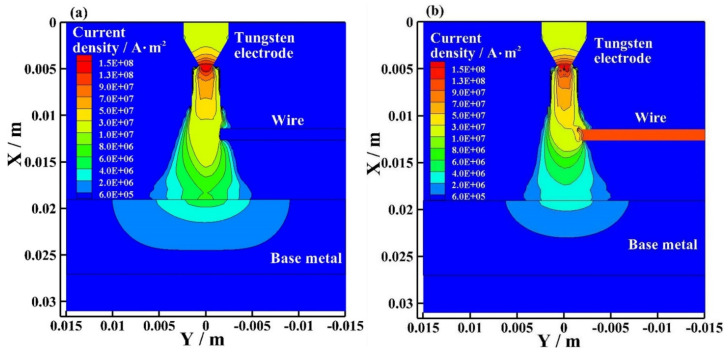
Current density distribution of bypass coupling arc at EN phase with and without bypass current, (**a**) 120 A and 0 A, (**b**) 60 A and 60 A.

Figure 11 shows the current density distribution of the bypass coupling arc at the EN phase with and without bypass current. As shown in Figure 11a, I_v_ = 120 A and I_b_ = 0 A. The wire exists, but the bypass arc does not exist. The maximum current density appears near the tip of the tungsten electrode. There is no current density distribution on the wire. The current density distribution is mainly concentrated on the plasma arc axis, and the maximum current density is 1.50 × 10^8^ A·m^−2^. As shown in Figure 11b, I_v_ = 60 A and I_b_ = 60 A. The bypass arc exists. The maximum current density appears near the tip of the tungsten electrode, and the maximum current density is 1.50 × 10^8^ A·m^−2^. The maximum current density on the wire is approximately 1.30 × 10^8^ A·m. In the bypass coupling arc with bypass curent, the current density distribution near the wire is more concentrated than the arc without bypass current at EN. The current density distribution on the base metal is smaller than the arc without bypass current at EN.

In the bypass coupling arc with bypass current, the wire separates a part of the current flowing through the base metal. The current density distribution of the bypass coupling arc on the base metal is smaller, and the current density distribution near the wire is more concentrated. The radius of the bypass coupling arc with bypass current action on the base metal is smaller; however, the current on the tungsten electrodes are all the same and the current density distribution near the tungsten electrode are all the same. The temperature distribution and flow vector distribution of the arc with and without bypass current are basically the same.

### 3.2. The Energy Transfer between Electrode and Coupling Arc

**Figure 12:**
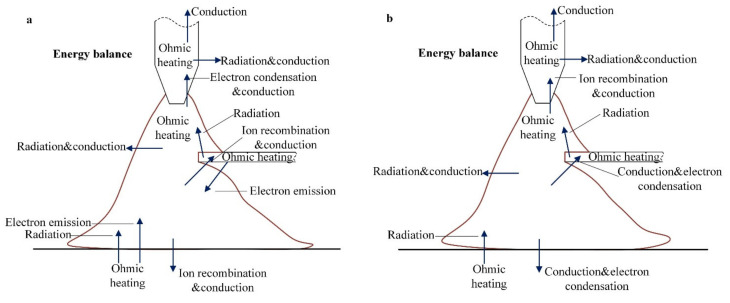
Schematic diagram of energy balance at EN and EP phases, (**a**) EP, (**b**) EN.

Figure 12 depicts the energy transfer between the arc and the electrodes. In this system, the energy transfer between the arc and electrodes (including base metal and wire) roughly includes electron heat, ion heat, thermal conduction, radiation loss, and joule heat [30]. The electron heat consists of electron condensation heating and thermionic electron emission cooling. The ion heat of the base metal and the wire only works in the EP phase. The electron and ion heat on the base metal and wire are mainly determined by I_v_ and I_b_. The thermal conduction of the arc to the base metal and the wire is determined by the temperature of the arc around the electrodes. The radiation loss is determined by material temperature. The joule heat is determined by the current flowing through the materials and the resistivity of the materials.

**Figure 13:**
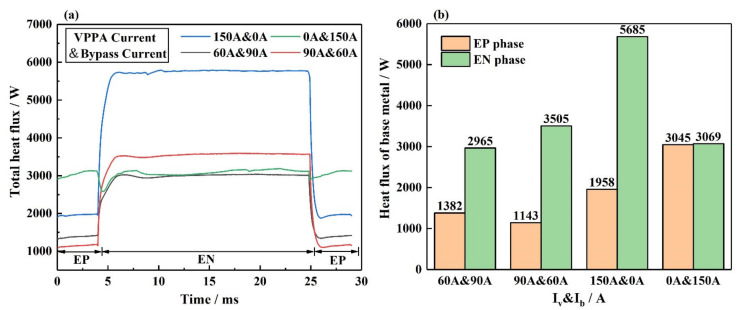
The total heat flux on the interface between bypass coupling plasma arc and base metal under different current conditions, (**a**) energy distribution over the period, (**b**) specific values of energy.

Figure 13a shows the total heat flux at the base metal. The sum of all the above energy transfer in Figure 12 is presented as the total heat flux. Figure 13b shows the specific values on the wire at the EN and EP phases. In I_v_ = 150 A and I_b_ = 0 A, the heat flux on the base metal is the largest at EN. In order to reduce the input of the base metal, the wire will separate a part of the current flowing through the base metal. For I_v_ = 90 A and I_b_ = 60 A and I_v_ = 60 A and I_b_ = 90 A, the heat flux on the base metal is smaller than that in I_v_ = 150 A and I_b_ = 0 A at the EN and EP phases. For I_v_ = 0 A and I_b_ = 150 A, the heat flux on the base metal is the largest at EP; the heat flux on the base metal at EN is also smaller than that in I_v_ = 150 A and I_b_ = 0 A. As the current on the base metal is 0 A, the base metal is heated only by the bypass arc. By comparison, the heat input to the base metal can be reduced by reducing the current flowing through the base metal. The bypass coupling plasma arc can realize the decoupling of heat transfer.

**Figure 14:**
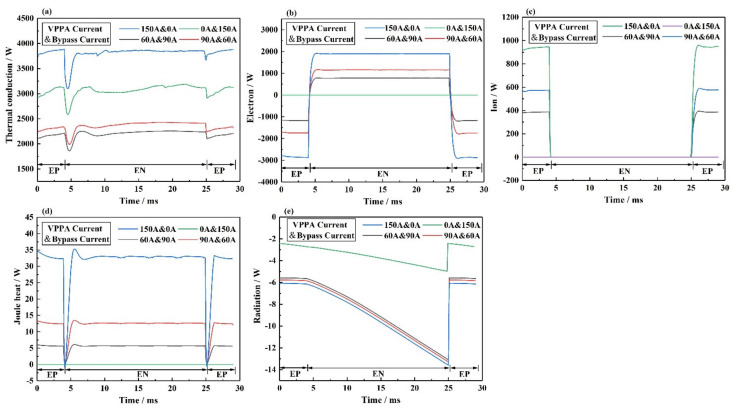
The energy transfer on the interface between bypass coupling plasma arc and base metal under different current conditions, including I_v_ = 150 A and I_b_ = 0 A, I_v_ = 0 A and I_b_ = 150 A, I_v_ = 60 A and I_b_ = 90 A, I_v_ = 90 A and I_b_ = 60 A, (**a**) thermal conduction, (**b**) electron, (**c**) ion, (**d**) joule heat, (**e**) radiation.

Figure 14 shows the thermal conduction, electron heat, ion heat, joule heat, and radiation loss on the interface between the bypass coupling plasma arc and base metal under different current conditions, including I_v_ = 150 A and I_b_ = 0 A, I_v_ = 0 A and I_b_ = 150 A, I_v_ = 60 A and I_b_ = 90 A, I_v_ = 90 A and I_b_ = 60 A. The sum of the above five energies is the total heat flux on the base metal. Figure 14a shows the thermal conduction in the interface between the plasma arc and base metal. In I_v_ = 150 A and I_b_ = 0 A, the thermal conduction on the base metal is the largest. After the wire separating a part of the current flowing through the base metal, the thermal conduction on the base metal is reduced. As I_v_ = 0 A and I_b_ = 150 A, the current on the base metal is 0 A. The base metal receives only thermal conduction from the bypass arc. Theoretically, the thermal conduction on the base metal should be the lowest, but the thermal conduction on the base metal is much higher than that in I_v_ = 90 A and I_b_ = 60 A or I_v_ = 60 A and I_b_ = 90 A. Figure 14b shows the electron heat in the interface between the plasma arc and base metal. In I_v_ = 0 A and I_b_ = 150 A, the I_v_ = 0 A, so the electron heat is 0 W. By comparing the other three curves, the ratio of electron heat is equal to the ratio of the current. This means that the electron heat transfer is completely decoupled. Figure 14c shows the ion heat on the interface between the plasma arc and base metal. In I_v_ = 0 A and I_b_ = 150 A, the I_v_ = 0 A, so the ion heat is 0 W. By comparing the other three curves, the ratio of electron heat is equal to the ratio of the current. This means that the ion heat transfer is also completely decoupled. Figure 14d shows the joule heat on the interface between the plasma arc and base metal. After the wire separating a part of the current flowing through the base metal, the change in joule heat is very small compared to the thermal conduction. Figure 14e shows the radiation loss on the interface between the plasma arc and base metal. The change in radiation loss is very small compared to the thermal conduction. By comparison, the thermal conduction on the base metal is not completely decoupled. This indicates that the bypass arc has a great influence on the heat input of the base metal. The system has not achieved complete thermal and mass decoupling.

**Figure 15:**
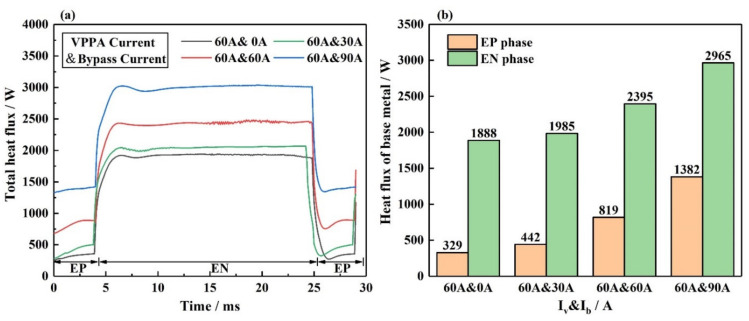
The total heat flux on the interface between bypass coupling plasma arc and base metal under different current conditions, including I_v_ = 60 A and I_b_ = 0 A, I_v_ = 60 A and I_b_ = 30 A, I_v_ = 60 A and I_b_ = 60 A, I_v_ = 60 A and I_b_ = 90 A, (**a**) energy distribution over the period, (**b**) specific values of energy.

Figure 15a shows the total heat flux at the base metal. The sum of all the above energy transfer in Figure 12 is presented as the total heat flux. Figure 15b shows the specific values on the base metal at the EN and EP phases. As shown in Figure 15b, it includes the I_v_ = 60 A and I_b_ = 0 A, I_v_ = 60 A and I_b_ = 30 A, I_v_ = 60 A and I_b_ = 60 A, I_v_ = 60 A and I_b_ = 90 A. The bypass current increases by 30 A each time. In the EP phase, from I_b_ = 60 A to I_b_ = 90 A, the increase in heat flux on the base metal is the largest. From I_b_ = 0 A to I_b_ = 30 A, the increase in heat flux on the base metal is the smallest. In the EN phase, from I_b_ = 60 A to I_b_ = 90 A, the increase i heat flux on the base metal is the largest. From I_b_ = 0 A to I_b_ = 30 A, the increase in heat flux on the base metal is the smallest. With the increase in the bypass current, the influence of the bypass arc on the base metal heat input increases.

**Figure 16:**
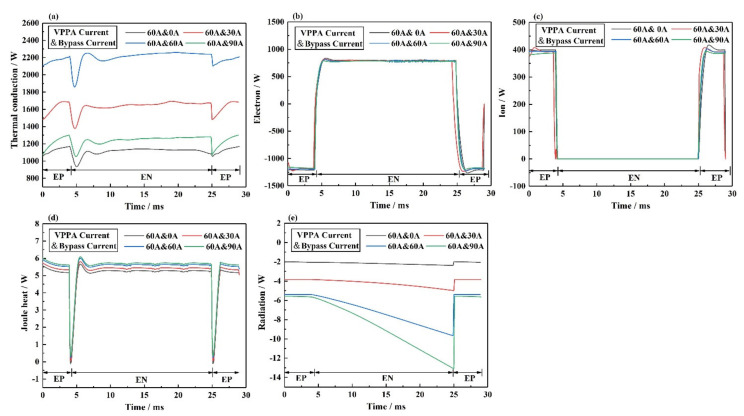
The energy transfer on the interface between bypass coupling plasma arc and base metal under different current conditions, including I_v_ = 60 A and I_b_ = 0 A, I_v_ = 60 A and I_b_ = 30 A, I_v_ = 60 A and I_b_ = 60 A, I_v_ = 60 A and I_b_ = 90 A, (**a**) thermal conduction, (**b**) electron, (**c**) ion, (**d**) joule heat, (**e**) radiation.

Figure 16 shows the thermal conduction, electron heat, ion heat, joule heat, and radiation loss on the interface between the bypass coupling plasma arc and base metal under different current conditions, including I_v_ = 60 A and I_b_ = 0 A, I_v_ = 60 A and I_b_ = 30 A, I_v_ = 60 A and I_b_ = 60 A, I_v_ = 60 A and I_b_ = 90 A. Figure 16a shows the thermal conduction in the interface between the plasma arc and base metal. In the EP phase, from I_b_ = 60 A to I_b_ = 90 A, the increase in thermal conduction on the base metal is the largest. From I_b_ = 0 A to I_b_ = 30 A, the increase in thermal conduction on the base metal is the smallest. In the EN phase, from I_b_ = 60 A to I_b_ = 90 A, the increase in thermal conduction on the base metal is the largest. From I_b_ = 0 A to I_b_ = 30 A, the increase in thermal conduction on the base metal is the smallest. Figure 16b shows the electron heat in the interface between the plasma arc and base metal. The current flowing through the base metal is constant, thus, the electron heat is constant. Figure 16c shows the ion heat on the interface between the plasma arc and base metal. The current flowing through the base metal is constant, thus, the electron heat is constant. Figure 16d shows the joule heat on the interface between the plasma arc and base metal. The change in joule heat is very small compared to thermal conduction. Figure 16e shows the radiation loss on the interface between the plasma arc and base metal. The change in radiation loss is very small compared to thermal conduction. The system has not achieved complete thermal and mass decoupling. The bypass arc affects the thermal conduction on the base metal, and with the increase in bypass current, the influence of the bypass arc on the base metal thermal conduction increases.

**Figure 17:**
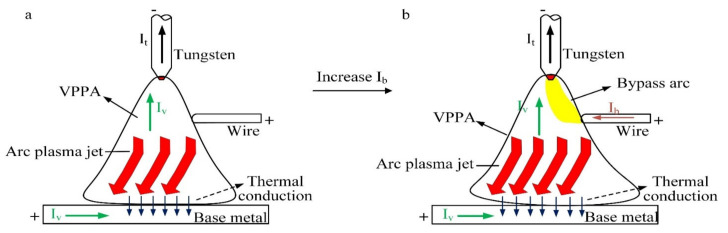
Schematic diagram of physics between bypass current variation and base metal heat input, (**a**) arc physics before bypass current changes, (**b**) arc physics after bypass current changes.

Figure 17 shows physics between the variation of the bypass current and heat input on the base metal. Figure 17a shows the bypass coupling variable polarity plasma arc without bypass current. Figure 17b shows the bypass coupling variable polarity plasma arc with bypass current. After increasing the bypass current, the bypass arc is produced. The current on the tungsten electrode increases and the temperature of the tungsten electrode increases. Because the tungsten electrode absorbs and emits electrons, the change in the tungsten electrode will affect the distribution of the arc. The charged plasma density in the whole space begins to increase as does the arc temperature. The heat input of the bypass coupling arc to the base metal increases, mainly concentrated on the increase in the thermal conduction between the arc and the base metal. Among these energy items of the base metal mentioned in Figure 12, the thermal conduction between plasma arc and base metal have a great impact on the base metal. Therefore, the increase in heat input on the base metal is mainly caused by the increase in thermal conduction. 

## 4. Conclusions

This work mainly explored the physical characteristics of the bypass coupling variable polarity plasma arc and the energy transfer between the arc and electrodes under different current conditions.

(1) The results show that the radius of the bypass coupling arc with or without bypass current action on the base metal is different, and the flow vector of the bypass coupling arc plasma with bypass current is larger than the arc without bypass current. The arc physics affect the heat transfer between the arc and base metal;
(2) By comparing the heat transfer on the electrodes’ boundary, it is found that this new coupling arc can control the decoupling of heat transfer between the arc and electrodes. However, the degree of heat transfer decoupling in the process is not 100%;
(3) In this process, the bypass arc affects the heat input of the base metal mainly by increasing thermal conduction to the base metal. The thermal conduction between the arc and base metal is mainly determined by the arc temperature. With the increase in the bypass current, the influence of the bypass arc on the base metal thermal conduction also increases.

These mechanisms revealed here are expected to realize the application of the bypass coupling variable polarity plasma arc in the welding or additive manufacturing field.

## Figures and Tables

**Table 1:** 5A06 aluminum properties and parameters.

		5A06 Aluminum
Thermal conductivity	*k* (w·m^−1^·k^−1^)	117
Specific heat	*c_p_* (J·kg^−1^·k^−1^)	921
Density	*ρ* (kg·m^−3^)	2.8
Electrical conductivity	*σ_e_* (S·m^−1^)	30
Liquidus temperature	*T_i_* (k)	913
Solidus temperature	*T_s_* (k)	873
Melting temperature	*T* (k)	1941

**Table 2:** Boundary conditions.

**Boundary**	***ν*** **/(m·s^−1^)**	***T*** **/** **K**	*φ*/V	*A*/(Wb·m^−1^)
*NM, LK*	$\nu =\nu_{constant}$	300	$\frac{\partial \phi }{\partial n}=0$	$\frac{\partial A}{\partial n}=0$
*PNO*	―	300	$-\sigma \frac{\partial \phi }{\partial n}=j_{given}$	$\frac{\partial A}{\partial n}=0$
*FTSRQOMVUED*	$\frac{\partial \nu }{\partial n}=0$	300	$\frac{\partial \phi }{\partial n}=0$	$\frac{\partial A}{\partial n}=0$
*ML, KY*	―	300	$\frac{\partial \phi }{\partial n}=0$	$\frac{\partial A}{\partial n}=0$
*ABC*	$\frac{\partial \nu }{\partial n}=0$	300	$\frac{\partial \phi }{\partial n}=0$	$\frac{\partial A}{\partial n}=0$
*BE*	$\frac{\partial \nu }{\partial n}=0$	300	$\frac{\partial \phi }{\partial n}=0$	*A* = 0

**Table 3:** Calculation conditions.

Case I_v_	I_b_	Plasma Gas Flow Rate	Shielding Gas Flow Rate	Standoff
1. 60 A	0 A	Ar: 2.0 L/min	Ar: 15 L/min	10 mm
2. 60 A	30 A	Ar: 2.0 L/min	Ar: 15 L/min	10 mm
3. 60 A	60 A	Ar: 2.0 L/min	Ar: 15 L/min	10 mm
4. 60 A	90 A	Ar: 2.0 L/min	Ar: 15 L/min	10 mm
5. 120 A	0 A	Ar: 2.0 L/min	Ar: 15 L/min	10 mm
6. 150 A	0 A	Ar: 2.0 L/min	Ar: 15 L/min	10 mm
7. 0 A	150 A	Ar: 2.0 L/min	Ar: 15 L/min	10 mm
